# Supplementary material for: Smoking-by-genotype interaction in type 2 diabetes risk and fasting glucose
Source: PLoS One. 2020 May 7;15(5):e0230815. doi: 10.1371/journal.pone.0230815 (PMC7205201; doi:10.1371/journal.pone.0230815)
Supplement: S1 Fig — (PDF) [file pone.0230815.s005.pdf]

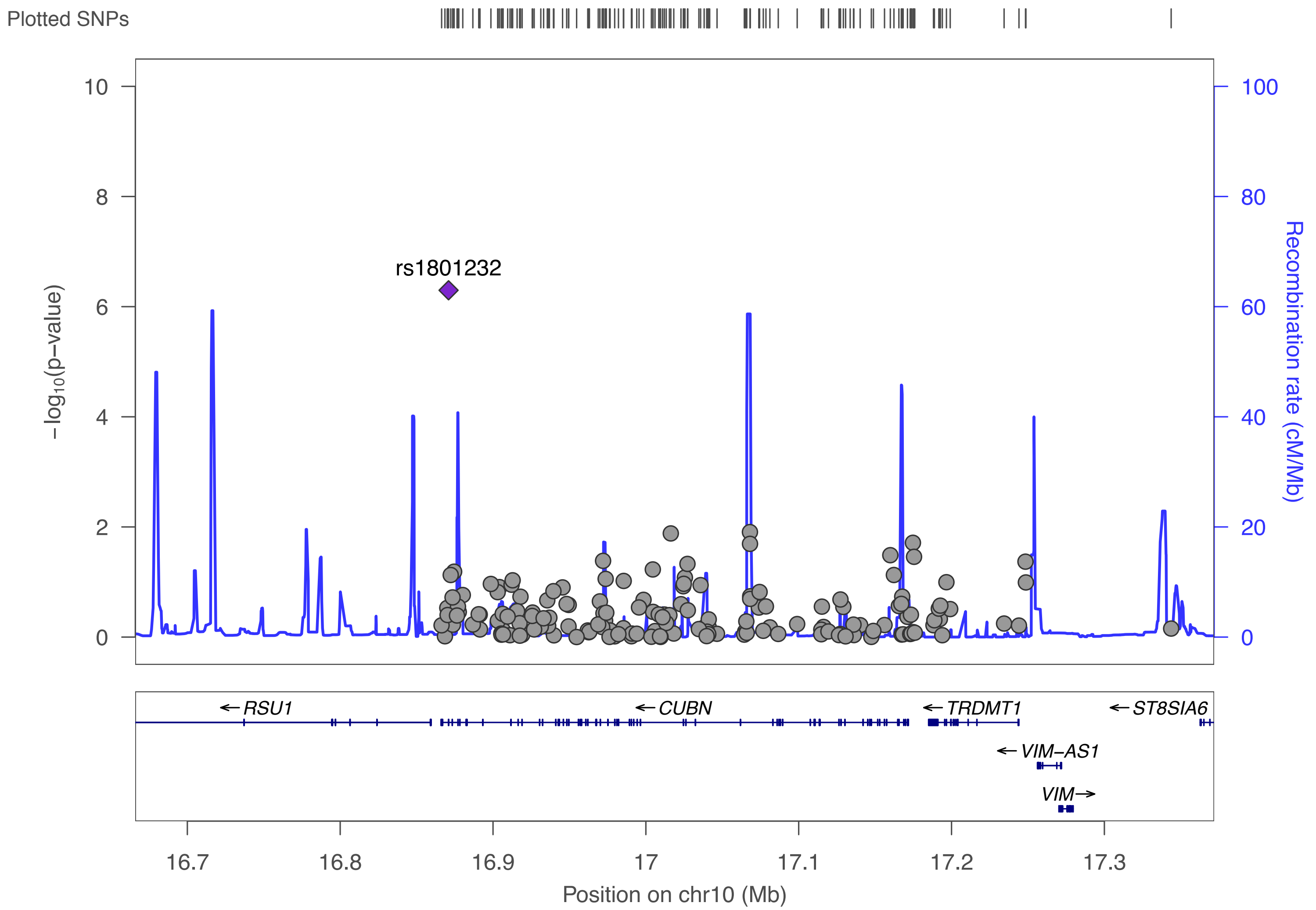

Supplemental Figure 1: Regional plot for rs1801232 with incident type 2 diabetes among smokers of African ancestry, indicating absence of linkage disequilibrium with other SNPs in YRI reference panel from the 1000 Genomes Project.
