# Supplementary material for: Smoking-by-genotype interaction in type 2 diabetes risk and fasting glucose
Source: PLoS One. 2020 May 7;15(5):e0230815. doi: 10.1371/journal.pone.0230815 (PMC7205201; doi:10.1371/journal.pone.0230815)
Supplement: Attachment — Submitted filename: Wu Smoking-T2D Response_to_Reviewers 2020303.docx [file pone.0230815.s006.docx]

To all reviewers: In revising the manuscript, we identified that one cohort’s data had been included in the T2D meta-analysis when it should have been included in the fasting glucose meta-analysis. We have redone the meta-analyses, as reflected in revised estimates in Table 1. The interpretation of our findings did not change as a result of this re-analysis.

**Reviewer #1: This is a paper investigating smoking by genotype interaction in incident T2DM and FG using data from two well-known consortia. The paper is well written and addresses a relevant topic.

There is something about this manuscript that puzzles me. In line 146-158 the authors briefly describe the cohorts from the two consortia contributing to this study, referring to Supplemental Table 1. According to the text, data from 5 cohorts were used for the discovery stage, and data from 14 cohorts in the replication stage.**

**In Supplemental Table 1, however, I noticed that for the replication stage, only 4 out of 14 cohorts contribute to the GEWIS for incident type 2 diabetes (row 12 in Supplemental Table 1: 10 do not have data on incident T2DM). In addition, only 12 cohorts contribute to the GEWIS for fasting glucose (2 do not have glucose measurements). This seemed odd to me, because the significant interactions observed in this study are for the analyses concerning incident T2DM, not for fasting glucose.....**

**The authors do not acknowledge this fact (4 instead of 14 cohorts for incident T2D in replications stage) anywhere in the manuscript. There may have been a mistake, an old version of Supplemental Table 1 may have been uploaded, or the results of the replications stage are really based on only 4 resp. 12 cohorts. If the latter is the case, then the authors should describe this early in the manuscript, because it puts the results in a completely different perspective. Now the authors only refer to their small number of incident T2D cases in the context of not being able to adjust for sex (line 197 and 422).**

Response: We thank the reviewer for this attention to detail. We have reviewed the cohort data, presented differently for illustration in this table:

|  | **Incident T2D** | **Baseline FG** |
| --- | --- | --- |
| **Discovery** | ARIC | ARIC |
|  | CARDIA | CARDIA |
|  | FHS | FHS |
|  | CHS | CHS |
|  | MESA | MESA |
| **Replication** |  | HyperGEN |
|  |  | HERITAGE |
|  |  | Generation Scotland |
|  | HANDLS | HANDLS |
|  |  | WHI |
|  | GENOA | GENOA |
|  | Rotterdam I | Rotterdam I |
|  | Rotterdam II | Rotterdam II |
|  | Rotterdam III | Rotterdam III |
|  |  | Erasmus Rucphen |
|  |  | Mount Sinai |
|  | WGHS |  |
|  |  | Family Heart Study |
|  | Jackson Heart Study | Jackson Heart Study |
|  |  | NEO |
|  | AGES | AGES |

All 5 discovery cohorts contributed data to both the T2D and FG analyses. Of the total 16 replication cohorts, 8 contributed data for the T2D analyses, and 15 contributed data for the FG analyses. We have now corrected this in the text, both in the Abstract (“up to 16 cohorts”) and the text in the Methods the reviewer mentioned.

**Have the authors performed a power analyses before conducting the study? If so, please include.**

Response: We have now added a power calculations section to the Methods section.

**In the cohort description it would really help to also list the number of incident cases of type 2 diabetes for the discovery and replication stage cohorts. It would also be informative to include the number of cohorts with family structures in discovery and replications stages.**

Response: We have added the number of T2D cases and controls to the text of the Results and to Supplemental Table 1, and we have added a new row indicating samples with independent or family structures in Supplementary Table 1.

**Why did the authors include the results from both the discovery and replication estimates in the meta-analysis, why not restrict this to replication results only?**

Response: We have presented all 3 analyses in Table 1, as is standard practice in GWAS: discovery set alone, replication set alone, and the combined discovery + replication sets.

**In the discussion a paragraph needs to be included about how the results and their meaning/interpretation are affected by the fact that sex could not be taken into account in most analyses.**

Response: Thank you for this comment. We have now further expounded this limitation in the Discussion.

**Minor comments**

**Regulome DB is not included in the methods section, but is included in Suppl. T4**

Response: We have now added a brief discussion and citation of RegulomeDB to the Methods.

**In line 269 please prove the 95% CI**

Response: Thank you for pointing out this omission. This has now been provided.

**In line 302 is reference is made to Table 1, but glucose results are not included in this Table.**

Response: Thank you. We have removed reference to Table 1 from that paragraph.

**For example, the refs 24 and 25 in line 337 are incorrect (refs 24 and 25 in the list (p. 23) do not cover the topic of B-cell dysfunction and apoptosis)**

Response: Thank you for bringing this to our attention. As a result, we have identified and corrected the numbering errors throughout the bibliography.

**Reviewer #2: Wu et al. have performed an array-wide association study for gene-smoking interactions on fasting glucose and incident T2D. They do not identify significant interaction effects, but report some novel significant associations when testing joint associations with SNP main effect and interaction, and associations that reach the significance in one stratum (smokers or ever smokers) but not the other. The paper includes some novel findings, but I have concerns about how the findings have been reported.

1. Defining interaction as a SNP main effect that is significant in only one smoking stratum (never or ever smokers) is misleading, as this does not give any information of how the SNP effect differs between the strata, i.e. the interaction effect. Please do not define these findings as interaction effects, but use appropriate wording.**

Response: We understand the reviewer’s point. In response, we have reworded the entire manuscript (Abstract, Introduction, Methods, Results, Discussion, captions, supplementary tables) to describe this as an endeavor to identify “potential gene-by-smoking interactions,” which we define in the Methods as follows:

“We defined a potential interaction effect between a locus and smoking if at least one of the following criteria was met: 1) significant SNP-by-smoking interaction; 2) significant joint 2-degree-of-freedom test of interaction and main effect, excluding SNPs with significant main effects; or 3) significant SNP effect in only one smoking stratum (never or ever smokers).”

We hope this careful, consistent wording and definition of terms allays the reviewer’s concern.

**2. It is also misleading to define the joint association of SNP main effect and interaction effect as an interaction. The identification of a locus in the joint test but not in a SNP main effect test could be due to an interaction effect, but could also be simply due to the adjustment of the model for smoking which increases power by explaining some of the residual variability in the outcome trait.**

Response: We have added this idea as a limitation of the analyses in the Discussion.

**3. Lines 214-215: “…excluding SNPs with significant main effects…” What P value was considered a significant SNP main effect? Considering that the TCF7L2 locus is the locus with the strongest known main effect on T2D risk, it is surprising that TCF7L2 was not excluded at this stage.**

Response: We thank the reviewer for this question. Significant main effect in the discovery analysis was considered p<10^-3^. In preparing our response to this question, we identified that one of the *TCF7L2* SNPs we originally reported (rs4074720) should not have been included. We have now removed that SNP from our significant findings and have corrected the abstract, results, Table 1, and discussion accordingly. We have also clarified the p<10^-3^ significance threshold in the Methods.

**4. Please do not only report results for single SNPs but indicate clearly when the SNPs represent independent loci, based on distance and/or LD threshold values, or conditional analyses.**

Response: After removing one *TCF7L2* SNP from our significant results (see #3 above), we now have 2 significant SNPs at *TCF7L2*. We now present conditional analyses of rs4132670 on the more significant SNP, rs12243326 (r^2^=0.72 and D'=0.95), and report that the former was no longer significantly associated with T2D.

**5. Lines 267-271: It is not possible that a SNP has a significant T2D-risk decreasing effect HR=0.64 in the discovery stage and a significant risk-increasing effect in the replication stage with HR=1.19, and the combined meta-analysis P value is still significant with HR=1.17. Please double-check this result. Due to the opposite direction of effect between the discovery and replication stages, the combined P value should be close to HR=1.**

Response: We have double-checked our analyses, and the numbers are correct. We have now added language to the Discussion highlighting the possibility that this finding is spurious: “However, the observation that the direction of effect was reversed in the replication and meta-analysis results compared with the discovery stage results calls into question the validity of this finding.”

**6. Line 269: “CI=XXX, XXX”, please add the missing numbers here.**

Response: We have corrected this omission.

**7. Line 355-356: Important to clarify whether the CKDGen consortium meta-analysis identified the same CUBN missense SNP as identified in the present study.**

Response: We have now performed conditional analyses of the *CUBN* SNP we identified on the previously reported SNP and found that our signal remains significant. We have now added these details to the Discussion section.

**8. Line 420: “such as” words are out of place here.**

Response: We have corrected this error.

**9. Lines 426-428: The conclusions are not justified by the data. This paragraph needs to be revised to be consistent with the findings. It should be clearly stated that no significant interaction effects were found. Rather, the authors found some loci that showed significant joint test associations for SNP main effect and interaction, and associations that were significant in one smoking stratum but not the other. Future studies with larger sample sizes are required to provide evidence of interaction for these loci.**

Response: We have now reworded the entire manuscript to describe our results as “potential interactions” and have defined that term. We have then added the reviewer’s concern to the Discussion: “Second, a locus identified by the inclusion of a significant joint test as one criterion for potential locus-smoking interaction may actually have a significant main effect, not a significant interaction with smoking, if the inclusion of smoking in the model explained residual variability in the outcome and increased power to detect main effects. To limit the impact of this misclassification, we excluded SNPs with significant main effects from eligibility for this criterion. Third, although we used data from about 75,000 individuals across the CHARGE Consortium Gene-Lifestyle Interactions Working Group to replicate our discovery analyses, data from larger cohorts such as the UK Biobank and Million Veteran Program now exist and might provide future opportunity for additional replication.”

**10. TABLE 1: It is critical to indicate which allele is the effect allele, and to add effect allele frequency in the table.**

Response: We have now indicated that “A1” is the coded effect allele and have added the A1 allele frequencies to Table 1.

**11. FIGURE 1d: The association pattern looks strange. What was the sample size and imputation quality for this variant? What reference panel was used for the LD?**

Response: The sample size for incident T2D among AA was 717, and the average imputation quality for rs1801232 was 0.913. For better organization, we have now limited Figure 1 just to rs1224336 in *TCF7L2* and moved the regional association plot for rs1801232 (*CUBN*) to Supplemental Figure 1, the caption of which now specifies that the 1000 Genomes Project YRI population was used as the reference.

**Reviewer #3: Wu et al. describe results from Gene x Smoking interactions analyses for type 2 diabetes (T2D) and fasting glucose (FG).

The study is based on a 2-stage design, including a discovery and replication stage. The discovery stage consists of 5 cohorts from the CARe consortium (N~24,000). The discovery samples were genotyped on the Custom ITMAT-Broad-CARe (IBC) genotyping array that contains ~50K SNPs selected for their impact on cardiovascular diseases. The replication stage consists of data from the CHARGE consortium (N~75,000). Both stages involve individuals of European and African ancestry (EA and AA, respectively). All analyses were stratified by ancestry status. Smoking was defined by a binary variable SMK that compares ever and never smokers.

For each ancestry group, each trait and each of the two stages, the authors applied four regression models: (i) An interaction model that includes SNP, SMK, SNPxSMK and other covariates, (ii) a main effect model (excluding the interaction term), (iii) a main effect model that was restricted to ever smokers and (iv) a main effect model that was restricted to never smokers. For each stage, the authors conducted fixed-effect meta-analysis to combine cohort-specific results. In addition, the results of the individual stages were meta-analysed.

They selected variants from discovery that showed (a) significant SNP x Smk interaction (Pint < 1e-3), (b) significant joint main+interaction effect (Pjoint <1e-3, excluding those with significant main effects Pmain <1e-3), or (c) significant subgroup effects (P<1e-3, excluding those that show an effect in the other subgroup as well). They selected 371 variants from discovery, which were then followed-up in a combined discovery+replication stage. They identified 6 SNPs for T2D with P<1e-7 for at least one of the three approaches (a)-(c). They did not identify any variant for FG. The identified variants are likely candidates for SNP x Smk interactions for T2D.

The study is informative and adds to previous work. It provides additional insight into the biological and genetic underpinning of T2D. The results will be of interest for those studying diabetes and related traits. I only have some minor comments:

1. Table 1 shows results for the 6 variants. It seems that only three of the 6 variants have nominal significant interaction P values. I think this deserves some discussion as to how much one can trust those interactions. Also, I would find it helpful to add P values for the test for difference between beta_never and beta_ever that may help the interpretation of these findings. The abstract says that the findings “provide evidence” for interactions. Personally, I would tone down on that a little bit. No doubt, the identified loci are likely candidates for interactions but not necessarily proven (as mentioned above, P int is not even nominal significant for three of the 6 variants).**

Response: In response to this and the critique from Reviewer 2, we have now reworded the entire manuscript (Abstract, Introduction, Methods, Results, Discussion, captions, supplementary tables) to describe this as an endeavor to identify “potential gene-by-smoking interactions,” which we specifically define in the Methods.

**2. Have the authors thought of testing the interaction for known main effect T2D and FG variants?**

Response: We thank the reviewer for this suggestion. We have now added that as a possible future direction for this work in the Discussion.

**3. Page 3, line 100: “cohorts”**

Response: We have corrected this typo.

**4. Page 11, line 269: CI’s are missing**

Response: We have now corrected the omission.
